# YOLO-PTHD: A UAV-Based Deep Learning Model for Detecting Visible Phenotypic Signs of Pine Decline Induced by the Invasive Woodwasp *Sirex noctilio* (Hymenoptera, Siricidae)

**DOI:** 10.3390/insects16080829

**Published:** 2025-08-09

**Authors:** Wenshuo Yang, Jiaqiang Zhao, Dexu Zhu, Zhengtong Wang, Min Song, Tao Chen, Te Liang, Juan Shi

**Affiliations:** 1 Beijing Key Laboratory for Forest Pest Control, Beijing Forestry University, Beijing 100083, China; 2 Sino-French Joint Laboratory for Invasive Forest Pests in Eurasia, Beijing Forestry University, Beijing 100083, China; 3 Shijiazhuang Institute of Fruit Trees, Hebei Academy of Agriculture and Forestry Sciences, Shijiazhuang 050061, China; 4 Heilongjiang Provincial Station for Forest Pest and Disease Control and Quarantine, Harbin 140080, China; 5 Fujin City Forest Pest and Disease Control and Quarantine Station, Jiamusi 146100, China

**Keywords:** invasive species, *Sirex noctilio*, deep learning, YOLO, UAV image, pine tree, object detection

## Abstract

*Sirex noctilio* is an invasive pest that contributes to pine tree decline, leading to visual symptoms such as needle discoloration, crown thinning, and eventual tree death. Detecting these visible phenotypic signs from drone imagery is challenging due to elongated or irregular crown shapes, weak color differences, and occlusion within dense forests. This study introduces YOLO-PTHD, a lightweight deep learning model designed for detecting visible signs of pine decline in UAV images. The model integrates three customized components: Strip-based convolution to capture elongated tree structures, Channel-Aware Attention to enhance weak visual cues, and a scale-sensitive dynamic loss function to improve detection of minority classes and small targets. A UAV-based dataset, the Sirex Woodwasp dataset, was constructed with annotated images of weakened, and dead pine trees. YOLO-PTHD achieved an mAP of 0.923 and an F1-score of 0.866 on this dataset. To evaluate the model’s generalization capability, it was further tested on the Real Pine Wilt Disease dataset from South Korea. Despite differences in tree symptoms and imaging conditions, the model maintained strong performance, demonstrating its robustness across different forest health scenarios. Field investigations targeting Sirex woodwasp in outbreak areas confirmed that the model could reliably detect damaged trees in real-world forest environments. This work demonstrates the potential of UAV-based visual analysis for large-scale phenotypic surveillance of pine health in forest management.

## Simple Summary

Pine trees are vital for forests and the environment, but they are increasingly threatened by an invasive insect called the *Sirex noctilio*. This pest weakens and kills trees, often without obvious early signs. Traditional inspection methods are slow and may miss early damage. In this study, we created a new method that uses UAVs and artificial intelligence to quickly find unhealthy pine trees by analyzing aerial images. Our approach can detect changes in needle color and tree shape, even in partially occluded scenes. We tested the method on real forest images and confirmed its accuracy through ground surveys. It worked well in different locations and with other tree diseases too. This technology offers a fast, accurate, and practical way to monitor forest health and could help prevent large-scale tree losses.

## 1. Introduction

Pine trees (*Pinus* spp.) are foundational elements of forest ecosystems worldwide, contributing essential ecological functions such as carbon sequestration, biodiversity maintenance, and watershed regulation. They also hold considerable economic and cultural value in many regions [1]. In recent decades, however, invasive pests and diseases, including pine wilt disease (*Bursaphelenchus xylophilus*) and the mountain pine beetle (*Dendroctonus ponderosae*), have caused widespread mortality in pine forests, disrupting ecosystem balance and reducing forest resilience [2,3]. Monitoring tree health status based on visible phenotypic changes is critical for assessing the impact of pests and guiding management. However, traditional ground-based surveys are constrained by dense canopy cover, limited site accessibility, and high labor demands, rendering them unsuitable for rapid, large-scale forest assessments.

Recent advances in unmanned aerial vehicle (UAV) platforms and deep learning algorithms have revolutionized forest health monitoring, particularly in the context of pest and disease surveillance [4,5,6,7]. UAVs enable high-resolution image acquisition across broad spatial extents, while convolutional neural networks (CNNs) have substantially improved the automated analysis of UAV imagery [8,9,10]. Among these, the YOLO (You Only Look Once) framework has become a widely adopted object detection algorithm in remote sensing due to its balance of speed and accuracy [11,12,13]. Recent research has highlighted the capability of YOLO-based object detection frameworks for identifying and localizing harmful pests in complex environmental scenes [14,15]. For example, DeepForest and YOLOv5 have been used to map bark beetle damage in Mexican pine stands [16], and an enhanced YOLO model (YOLO-PWD) has improved pine wilt disease detection by distinguishing discolored and dead trees with higher precision [17].

The invasive woodwasp *Sirex noctilio* (Hymenoptera, Siricidae), a xylophagous insect native to Eurasia and North Africa, has become an invasive pest of increasing concern in pine ecosystems worldwide. It is now recognized by the Food and Agriculture Organization (FAO) as a high-priority forest quarantine pest due to its destructive capacity and rapid spread [18]. Female Sirex woodwasps deposit eggs into weakened or stressed pine trees, simultaneously injecting a phytotoxic mucus and spores of the symbiotic fungus *Amylostereum areolatum*. The synergistic action of these agents disrupts vascular function and leads to tree mortality [19,20,21]. Although pine wilt disease (PWD) is more widely known, the ecological threat posed by Sirex woodwasp infestations is comparably severe and warrants equal attention [22].

In China, this species was first reported in 2013 in plantations of *Pinus sylvestris* var. *mongholica* in Heilongjiang Province, where it has since become a major forest health concern. Several remote sensing–based methods have been proposed to detect infestations [23,24]. For example, spectral indices derived from multispectral imagery and photometric point clouds have been used in machine learning frameworks [25]; hyperspectral data combined with Random Forest (RF) and Support Vector Machine (SVM) models have been applied to distinguish infested from healthy or lightning-damaged trees [26]; and RF-based classification using PlanetScope imagery has shown promising accuracy in mapping damage [27]. These approaches have contributed valuable insights but still face notable limitations, such as reliance on handcrafted features, low spatial resolution, and poor generalization under complex forest conditions.

Previous ecological studies have established that the Sirex woodwasp preferentially infests weakened or drought-stressed pine trees [28,29,30]. These trees typically exhibit subtle indicators of decline, such as slight needle yellowing, reduced foliage density, and crown thinning, making it difficult to distinguish them from healthy trees [31]. Traditional remote sensing methods attempt to capture these symptoms through handcrafted spectral or geometric features, followed by classification using algorithms such as Random Forest and Support Vector Machine [32,33]. However, these handcrafted features often fail to represent the nuanced changes in color and canopy structure that signal early stages of infestation.

Deep learning offers a more robust solution to this challenge. CNNs can automatically learn hierarchical feature representations from high-resolution UAV imagery, enabling improved classification performance and stronger generalization across varied forest environments [34,35]. Such models are capable of capturing spatial patterns in needle coloration and canopy morphology that are indicative of incipient Sirex woodwasp damage. Nevertheless, detecting these weak visual cues remains difficult in practice due to confounding factors such as background vegetation, inconsistent lighting, variable image resolution, and the high aspect ratio of pine tree crowns. The present study addresses these challenges by designing a specialized deep learning architecture tailored to detecting early signs of pine tree health decline in UAV imagery under complex field conditions.

Although previous studies have applied remote sensing and machine learning techniques to detect pine wilt disease and other forest threats, no research to date has explored the use of deep learning methods for detecting and classifying pine trees damaged by Sirex woodwasp. Addressing this gap, the present study leverages the known ecological characteristics of the Sirex woodwasp, particularly its preference for weakened or stressed pine hosts, to develop a robust deep learning framework for pine tree health assessment based on UAV imagery. We propose a novel model, YOLO-Pine Tree Health Detection (YOLO-PTHD), designed to classify individual trees as healthy, weakened, or dead, to support early detection of Sirex woodwasp damage. Unlike YOLO-PWD [17], which analyzes medium-resolution UAV imagery captured at higher flight altitudes for large-area monitoring, YOLO-PTHD is optimized for high-resolution images from lower-altitude flights, enabling finer detection of early phenotypic signs such as needle discoloration and crown thinning. To support this, YOLO-PTHD integrates strip convolution, context anchor attention, and a dynamic loss function, enhancing detection of elongated crowns and partially occluded trees. The main contributions of this study are as follows:

1. We constructed a new UAV-based dataset of pine trees infested by Sirex woodwasp, including both orthophotos and oblique-angle images collected from field-verified outbreak areas.
2. We designed YOLO-PTHD by incorporating three key architectural components: a strip convolutional structure using separate vertical and horizontal filters to accommodate the elongated crown morphology of pine trees; a context anchor attention mechanism that captures long-range spatial dependencies to improve distinction between healthy and infested trees; and a dynamic loss function that adjusts adaptively based on tree size, improving both localization and classification accuracy.
3. We conducted a series of ablation experiments to evaluate the performance contribution of each module, supported by qualitative visualization of model attention on canopy and needle features.
4. We validated the model against field survey data and demonstrated a high detection accuracy of 96.3% in identifying weakened trees damaged by Sirex woodwasp.
5. We evaluated the model’s generalization ability using the publicly available the Real PWD dataset from South Korea, which confirmed that YOLO-PTHD can be effectively applied to detect symptoms caused by other invasive pests and diseases. These findings highlight the practical value and transferability of the proposed model in large-scale pine forest health monitoring.

## 2. Materials and Methods

### 2.1. Survey Region

**Figure 1:**
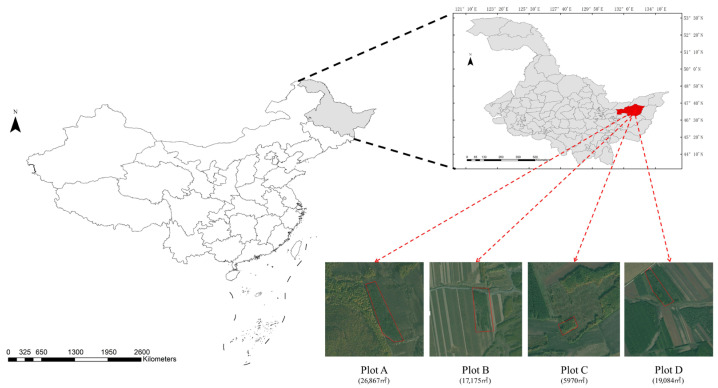
Geographical location of the study area and satellite imagery of the plots. Plot A (26,867 m^2^), Plot B (17,175 m^2^), Plot C (5970 m^2^), and Plot D (19,084 m^2^) are all depicted. The map legend indicates the scale used and the units of measurement.

The study site (Figure 1) is located in Fujin City, Jiamusi City, Heilongjiang Province, in northeastern China, with geographical coordinates ranging from 131°25′ to 133°26′ East longitude and 46°45′ to 47°45′ North latitude. Fujin City has a temperate continental monsoon climate, characterized by an average annual temperature of 3.6 °C and an average annual precipitation of approximately 339.5 mm. This study focuses on two main areas with four plots, all of which have experienced large-scale outbreaks of the Sirex woodwasp. The pine species damaged by the Sirex woodwasp in this study is *Pinus sylvestris* var. *mongholica*. Plot A consists of a mixed coniferous and broadleaf forest of *Pinus sylvestris* var. *mongholica*. that is 30 to 40 years old, while the other plots are pure stands of the same pine species, aged between 40 and 50 years.

### 2.2. Data Collection and Preprocessing

#### 2.2.1. UAV-Based High-Resolution Image Collection

In this study, a DJI Mavic 3 UAV platform (DJI, Shenzhen, China) equipped with a Hasselblad L2D-20c aerial photography camera was used to collect high-resolution images. Flight operations were conducted using the DJI Fly app for Android (version 1.15.8) , with multiple flight missions carried out from June to August 2024. Two photography techniques were employed: orthogonal and oblique photography. UAVs were used to conduct multi-angle orthogonal and oblique photography to capture high-resolution images of weakened and dead trees. A total of 1751 images were collected using both methods, with each image having a resolution of 5280 × 2970. Table 1 provides detailed information on the image collection for the four plots.

#### 2.2.2. Ground Survey and Validation Data Collection

**Figure 2:**
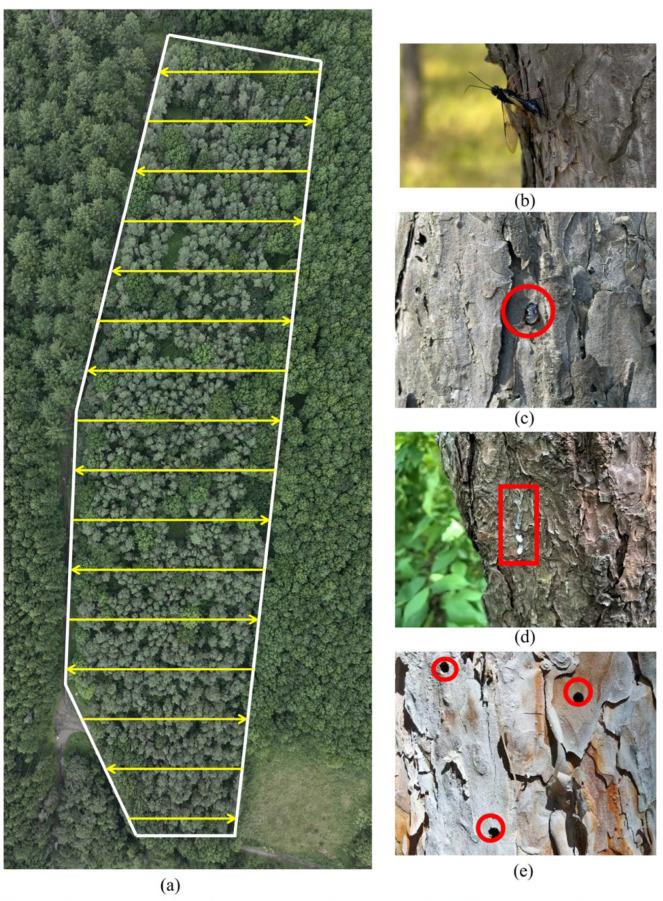
The UAV flight path and ground survey. (**a**) UAV flight trajectory; (**b**) pine trees damaged by Sirex woodwasp (oviposition sites); (**c**) tear-shaped resin beads at egg deposition sites (transparent appearance, indicating newly damaged trees); (**d**) tear-shaped resin beads at egg deposition sites (white, dried resin droplets, indicating previously damaged trees); (**e**) exit holes (3–10 mm in diameter) left by emerging Sirex woodwasp adults.

In coordination with the ground survey, UAV flight missions and field surveys were conducted from September to October 2024. The Rainbow Cloud Software program (version 3.85) was used to plan flight paths for image acquisition over Plot A, covering 26,867 m^2^ with 141 waypoints along 16 main flight lines. An 80% overlap in both the along-track and cross-track directions, a flight speed of 3 m/s, and a flight altitude of 40 m were maintained to ensure comprehensive orthophoto coverage of Plot A. Figure 2a illustrates the flight path, which resulted in the acquisition of 141 high-resolution images. The entire UAV flight mission took approximately 90 min to complete the aerial survey of Plot A.

Simultaneously, six trained researchers conducted a comprehensive ground survey of pine trees infested by Sirex woodwasp across the entire Plot A area. The purpose of this survey was to validate the model’s ability to accurately identify pine trees damaged by Sirex woodwasp. Given that the emergence and oviposition period of Sirex woodwasp typically occurs between August and September each year [19,36], the ground survey was carried out over a one-month period from September to October 2024.

The field team strictly followed the identification standards illustrated in Figure 2b–e, working 8 h per day. It took 12 consecutive days to complete the survey of Plot A. Figure 2b displays images of newly emerged female Sirex woodwasp depositing eggs on tree trunks. During the ground survey, the researchers focused on both current-year and previous-year damage caused by Sirex woodwasp. To distinguish between these two types of damage, the researchers primarily relied on the characteristics of tear-shaped resin produced by the trees [37]. Figure 2c illustrates the state of newly formed tear-shaped resin, which appears transparent and has just been secreted from oviposition sites [38]. Figure 2d shows the state of older tear-shaped resin, which has dried into a white color and exhibits a downward flow pattern. Additionally, the presence of exit holes (3 mm to 10 mm in diameter) left by Sirex woodwasp on tree trunks served as evidence to determine whether the trees had previously been infested [39,40], as shown in Figure 2e.

#### 2.2.3. UAV Image Preprocessing

To ensure the accuracy of the dataset, this study conducted preprocessing on the collected images. The researchers excluded images that did not contain weakened or dead pine trees, as well as those with an excessive presence of other broadleaf species and crops (where the proportion of these species exceeded 90% of all trees in the image). Additionally, images that were blurry or had poor visibility were also excluded. After preprocessing of the images, a total of 1330 image files were retained Table 1.

#### 2.2.4. Health Classification System and Labeling Solution

Research indicates that the Sirex woodwasp predominantly targets weakened pine trees [20,36,41]. In this study, following ground surveys conducted by researchers, the preprocessed images were evaluated. According to previous relevant studies, the health status of all pine trees were categorized into three classes: healthy, weakened, and dead. The criteria for these classifications are as follows:

1. Healthy: Needles are a deep green color and have a glossy appearance; the tree is overall lush and full, with a complete, uniform, and dense canopy. Branch growth is robust, and there are no notable signs of disease or pest damage [42,43].
2. Weakened: Needles are beginning to yellow and wither; the canopy is sparse and appears less dense compared with healthy trees. There are signs of death in the top branches, along with some needle loss [31,44].
3. Dead: All needles are completely dried up or have fallen off, leaving no green parts remaining. The canopy is nearly leafless and appears bare, with most branches dead and a dull color that indicates no signs of life [45].

This classification reflects the visible phenotypic responses of pine trees to Sirex woodwasp attack.

#### 2.2.5. Image Annotation and Dataset Establishment

**Figure 3:**
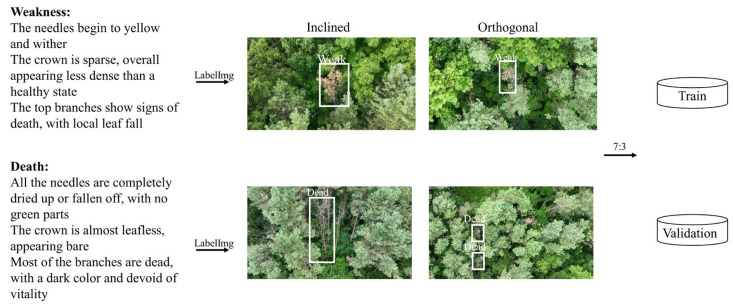
Process of establishing a dataset.

Researchers used the annotation tool LabelImg [46] to classify the health conditions of pine trees in the preprocessed images. Following the health assessment criteria outlined in Section 2.2.4, weakened trees were labeled as “Weak,” and dead trees as “Dead.” Figure 3 illustrates the image annotation process, showing collected images from both orthophoto and oblique angles of damaged trees. The researchers outlined weakened and dead trees that met the criteria and assigned the labels “Weak” and “Dead” using the Create RectBox function of the LabelImg (version 1.8.1).

All annotated images and corresponding annotation files were randomly split into training and validation sets in a 7:3 ratio. This partitioning is crucial for training deep learning models to accurately classify tree health status in UAV imagery. Additionally, the Real Pine Wilt Disease (R-PWD) dataset proposed by [47] was used in this study to validate the generalization capability of the model in detecting pine wilt disease (Table A1).

### 2.3. Deep Learning Model

Detecting weakened and dead pine trees in high-resolution images is a significant challenge. This requires both precise pine tree localization and accurate health status differentiation. An effective detection algorithm is essential for evaluating pine tree health.

This study introduces YOLO-Pine Tree Health Detection (YOLO-PTHD), a novel model for detecting pine tree health conditions in high-resolution imagery. The model is built on the YOLOv11 framework proposed in [48]. YOLOv11 improves upon YOLOv8 [49] by integrating C3k2, C2PSA, and DWConv, enhancing model performance. This study introduces targeted innovations in the backbone, neck, and head to enhance pine tree health status identification.

#### 2.3.1. Enhancements to Strip Feature Extraction in the Backbone Section

**Figure 4:**
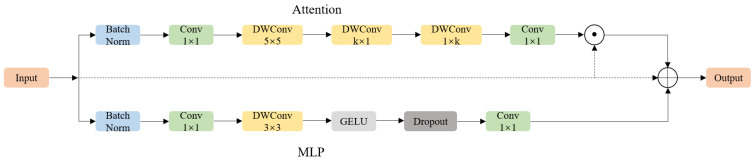
C3k2_Strip structure diagram. ⊙ denotes element-wise multiplication, and ⊕ denotes element-wise addition. Solid lines indicate the main data flow, while dashed lines indicate residual connections.

In this study’s dataset, weakened and dead pine trees have high aspect ratios and similar image area proportions to surrounding healthy trees. We propose a StripBlock-enhanced [50] variant of the YOLOv11 backbone, termed C3k2_Strip, to enhance the model’s ability to extract features of pine trees with high aspect ratios. Each C3k2_Strip block replaces the standard convolutional sub-blocks with a custom-designed StripBlock, which integrates two lightweight components: Strip Attention and Strip MLP. Figure 4 illustrates the detailed structure of the C3k2_Strip module.

The Strip Attention module applies a series of directional depthwise convolutions, including vertical (*k* × 1), horizontal (1 × *k*), and an initial spatial aggregation via (5 × 5) depthwise convolution. This design enables the model to extract long-range features along dominant axes. In our setting, we fix *k* = 10 to achieve a balance between receptive field and efficiency. The full computation can be formulated as follows:

(1) $$\begin{array}{c} Attn\left(x_{1}\right)=x\cdot {\mathrm{Conv}}_{1\times 1}\left({\mathrm{DWConv}}_{k\times 1}\left({\mathrm{DWConv}}_{1\times k}\left({\mathrm{DWConv}}_{5\times 5}\left(x_{1}\right)\right)\right)\right) \end{array}$$

To complement spatial modeling, the Strip MLP module performs inter-channel mixing through two pointwise (1 × 1) convolutions and an intermediate depthwise convolution. This preserves locality while improving channel capacity:

(2) $$\begin{array}{c} MLP\left(x_{1}\right)={\mathrm{Conv}}_{1\times 1}^{\left(2\right)}\left(\mathrm{Drop}\left(\mathrm{GELU}\left({\mathrm{DWConv}}_{3\times 3}\left({\mathrm{Conv}}_{1\times 1}^{\left(1\right)}\left(x_{1}\right)\right)\right)\right)\right) \end{array}$$

The output of the StripBlock follows a dual residual formulation, with learnable layer scaling coefficients $\gamma_{1}$ and $\gamma_{2}$, to enhance stability during training:

(3) $$\begin{array}{c} \hat{x}=x_{1}+\gamma_{1}\cdot Attn\left({\mathrm{BN}}_{1}\left(x_{1}\right)\right)+\gamma_{2}\cdot MLP\left({\mathrm{BN}}_{2}\left(x_{1}\right)\right) \end{array}$$

**Figure 5:**
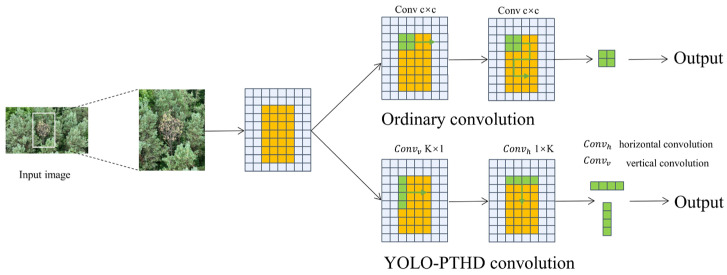
Comparative illustration of convolution operations.

As shown in Figure 5, a comparison between standard convolution and the proposed strip-based convolution approach. Traditional convolution uses square kernels (e.g., c × c) to scan the image and extract local features. In contrast, the proposed YOLO-PTHD convolution decomposes the process into two orthogonal strip convolutions: vertical (*k* × 1) and horizontal (1 × *k*). This directional design enables more effective feature extraction along elongated structures such as pine tree trunks and branches, which often exhibit high aspect ratios.

#### 2.3.2. Enhancements to the Context Anchor Attention Mechanism in the Neck Section

In pine forests, weakened and dead pine trees resemble healthy ones in canopy and branch structure. Additionally, in a single image, weakened and dead pine trees occupy a smaller proportion, while healthy pine trees and other vegetation dominate. Therefore, distant but related information, such as other vegetation, is crucial for accurate identification and differentiation.

To address this challenge, we replace the standard C3k2 block with a novel attention-enhanced variant, termed C3k2_CAA [51] to improve the neck’s capacity to integrate long-range contextual features from various backbone stages. This module integrates multiple Channel-Aware Attention (CAA) blocks to improve feature recalibration in both spatial and channel dimensions.

The CAA module adopts a squeeze-and-direction strategy: a global average pooling first captures contextual priors, followed by a sequence of directional depthwise convolutions to encode orientation-specific responses. In our implementation, *k* = 10 is used for both horizontal and vertical kernels. The process is expressed as follows:

(4) $$\begin{array}{c} \mathrm{CAA}\left(x_{2}\right)=x\cdot \sigma \left(\mathrm{Con}v_{1\times 1}^{\left(2\right)}\left(\mathrm{DWCon}v_{k\times 1}\left(\mathrm{DWCon}v_{1\times k}\left(\mathrm{Con}v_{1\times 1}^{\left(1\right)}\left(\mathrm{AvgPoo}l_{7\times 7}\left(x_{2}\right)\right)\right)\right)\right)\right) \end{array}$$

This mechanism generates direction-aware channel-wise weights, enabling fine-grained attention over elongated objects. The overall C3k2_CAA block integrates multiple such attention units and concatenates them via pointwise fusion:

(5) $$\begin{array}{c} y={\mathrm{Conv}}_{1\times 1}\left(\mathrm{Concat}\left(x_{2},{\mathrm{CAA}}_{1}\left(x_{2}\right),\ldots ,{\mathrm{CAA}}_{n}\left(x_{2}\right)\right)\right) \end{array}$$

**Figure 6:**
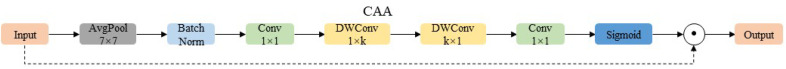
C3k2_CAA structure diagram. ⊙ denotes element-wise multiplication. Solid lines indicate the attention computation path, while the dashed line represents the skip connection that bypasses the attention computation and feeds the original features directly to the multiplication node.

This design enhances the neck’s feature fusion ability and benefits dense object representation learning. Figure 6 shows the internal structure of the C3k2_CAA module.

**Figure 7:**
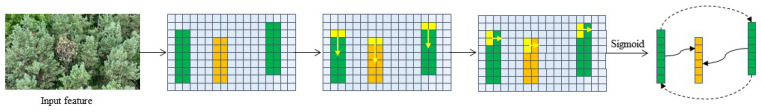
Schematic of the Channel-Aware Attention (CAA) mechanism.

As shown in Figure 7, the proposed Channel-Aware Attention (CAA) unit first performs a horizontal (*k* × 1) depthwise convolution and then a vertical (1 × *k*) depthwise convolution. This sequence extracts orientation-aware features, after which a sigmoid gate re-weights the input map to fuse long-range contextual cues and sharpen deep feature representation.

#### 2.3.3. Dynamic Loss Function Enhancements in the Head Section

Analyzing the dataset revealed significant variations in pine tree sizes in images captured through orthogonal and oblique photography. Due to variations in the angle between the UAV-mounted camera and the trees, Figure 3 illustrates that the dimensions of damaged pine trees in orthophotos are significantly smaller than those in oblique images, while also failing to meet the aspect ratio characteristics.

To overcome the degraded localization performance caused by small, low-aspect-ratio targets in orthophotos, we adopt the SDIoU loss function [52]. Unlike CIoU, SDIoU dynamically adjusts the penalty strength based on the predicted box’s size and shape, offering stronger supervision for small or irregular-shaped objects. This enhancement improves bounding box regression accuracy in scenes with orthogonal-view distortion, dense target distribution, and varied object scales.

Given a predicted bounding box $b_{1}$ with width $w_{1}$ and height $h_{1}$, and a target box $b_{2}$ with width $w_{2}$ and height $h_{2}$, their respective areas are $\mathrm{area}\left(b_{1}\right)$ = $w_{1}$*⋅*$h_{1}$ and $\mathrm{area}\left(b_{2}\right)$ = $w_{2}$*⋅*$h_{2}$. The basic IoU is calculated as follows:

(6) $$\begin{array}{c} IoU\left(b_{1},b_{2}\right)=\frac{\mathrm{area}\left(b_{1}\cap b_{2}\right)}{\mathrm{area}\left(b_{1}\right)+\mathrm{area}\left(b_{2}\right)-\mathrm{area}\left(b_{1}\cap b_{2}\right)} \end{array}$$

To better align the aspect ratios of the predicted and target boxes, SDIoU incorporates a shape penalty term v, defined as follows:

(7) $$\begin{array}{c} v=\frac{4}{\pi^{2}}{\left(\arctan\frac{w_{2}}{h_{2}}-\arctan\frac{w_{1}}{h_{1}}\right)}^{2} \end{array}$$

In addition, SDIoU introduces two adaptive coefficients, the aspect ratio alignment factor α and the size-aware penalty coefficient β, which are computed as follows:

(8) $$\begin{array}{c} \alpha =\frac{v}{1-\mathrm{IoU}+v},\;\beta =\delta \cdot \left(1-\frac{\log\left(1+\mathrm{area}\left(b_{2}\right)\right)}{\log\left(1+S_{max}\right)}\right) \end{array}$$

Here, δ = 0.5 is a fixed coefficient used to balance the loss components and control the maximum penalty strength [52]. The parameter $S_{max}$ is set to 90 × 90 in this study, corresponding to the largest labeled target in orthophotos. This choice ensures that small targets—frequent in orthogonal views—receive strong supervision via β ≈ δ, while large targets in oblique views lead to β → 0, thereby avoiding excessive penalization. The log-based formulation enables smooth, scale-adaptive loss modulation across diverse object sizes without requiring additional tuning.

The complete SDIoU loss integrates IoU, center distance $\rho^{2}$, shape penalty v, and the size-aware coefficient β, and is formulated as follows:

(9) $$\begin{array}{c} SDIoU=\delta -\beta +\left(1-\delta +\beta \right)\left(\mathrm{IoU}-\alpha v\right)-\left(1+\delta -\beta \right)\frac{\rho^{2}}{c^{2}} \end{array}$$

where $\rho^{2}$ is the squared distance between the centers of $b_{1}$ and $b_{2}$, and $c^{2}$ is the squared diagonal length of the smallest enclosing box covering both $b_{1}$ and $b_{2}$.

**Figure 8:**
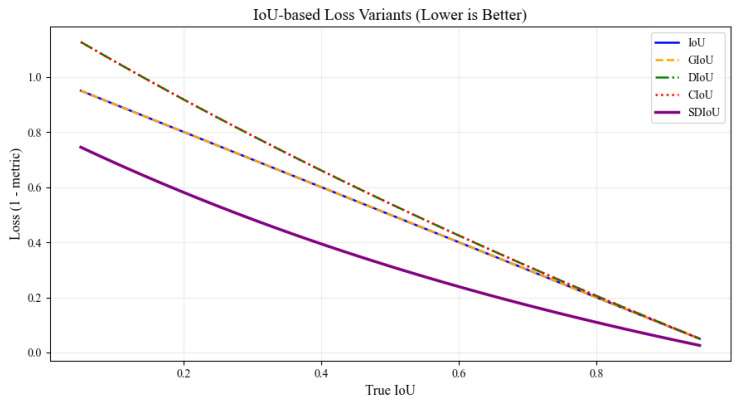
Comparison of IoU-based loss variants.

This formulation improves regression quality by penalizing size and shape mismatches, especially in dense scenes and small object settings. As visualized in Figure 8, SDIoU consistently produces lower loss values compared to traditional IoU-based variants, particularly in low-IoU scenarios where small or irregular objects dominate. This dynamic adjustment makes SDIoU more effective for challenging detection tasks involving dense scenes, varied scales, and distorted bounding boxes.

#### 2.3.4. YOLO-PTHD Network Architecture and Training Parameters

**Figure 9:**
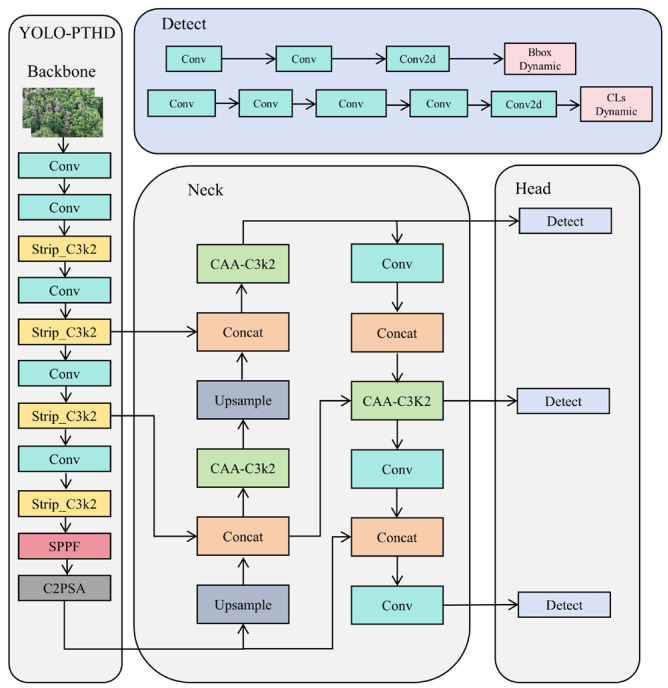
YOLO-PTHD network architecture.

Figure 9 illustrates the network architecture of the proposed YOLO-PTHD model, comprising three core components: Backbone, Neck, and Detect. These three modules—StripBlock, CAA, and SDIoU—jointly form the core of YOLO-PTHD’s design.

The improved YOLO-PTHD model extracts target pine tree features via the Backbone, fuses multi-stage Backbone features in the Neck using contextual anchor information, and dynamically adjusts scale and localization loss influence in the Detect module based on target tree size, thereby enhancing performance.

The training hyperparameters used for model optimization are summarized in Table 2. All training is conducted on a Windows 11 operating system, equipped with 32 GB of RAM, a 13th Gen Intel(R) Core(TM) i7-13700KF CPU running at 3.40 GHz, and an NVIDIA GeForce RTX 4070 Ti 12 GB GPU.

### 2.4. Evaluation Metrics

To evaluate the performance of the proposed model, this study employs the F1 score, and mAP as metrics for detection performance assessment. Additionally, the computational requirements of the trained model are evaluated using giga floating point operations (GFLOPs) quantify the demand on hardware computational resources. The calculation of detection performance metrics relies on the precision (*P*) and recall (*R*) indicators.

(10) $$\begin{array}{c} P=\frac{\mathrm{TP}}{\mathrm{TP}+\mathrm{FP}} \end{array}$$

(11) $$\begin{array}{c} R=\frac{\mathrm{TP}}{\mathrm{TP}+\mathrm{FN}} \end{array}$$

(12) $$\begin{array}{c} F1=\frac{2P\cdot R}{P+R} \end{array}$$

(13) $$\begin{array}{c} mAP=\frac{1}{N}\sum_{i=1}^{N}\left(\int_{0}^{1}P_{i}\left(R\right)\;dR\right) \end{array}$$

where true positive (TP) refers to the number of positive samples that the model accurately identifies as positive. False positive (FP) indicates the number of negative samples that the model incorrectly classifies as positive. False negative (FN) represents the number of positive samples that the model incorrectly identifies as negative. N denotes the total number of categories.

In addition to evaluating the overall detection performance of the YOLO-PTHD model, this study also aims to assess its capability to specifically detect pine trees weakened by Sirex woodwasp. To this end, we introduced an evaluation metric termed accuracy rate (AR).

The AR is defined as the proportion of damaged pine trees, as identified through ground surveys, that were successfully detected by the model:

(14) $$\begin{array}{c} \mathrm{AR}\%=\left(\frac{SD}{DT}\right)\times 100 \end{array}$$

where DT represents the total number of damaged trees recorded during the ground survey, and SD denotes the number of those trees that were correctly identified by the model as weakened. This metric provides a targeted evaluation of the model’s effectiveness in identifying trees specifically affected by Sirex woodwasp, which is crucial for forest health monitoring and pest outbreak prevention efforts.

## 3. Results

This section presents a comprehensive evaluation of the proposed YOLO-PTHD model. First, we assess its performance on the Sirex woodwasp (SW) dataset specifically constructed in this study, including detection accuracy across different health categories, ablation studies to examine the contribution of each architectural module, and validation using ground survey data. These experiments collectively demonstrate the model’s effectiveness in detecting pine trees affected by Sirex woodwasp under real-world conditions.

To further evaluate the model’s generalization capability, we introduce the Real Pine Wilt Disease (R-PWD) dataset, described in Appendix A. Two experimental settings are explored: (i) training and testing on the R-PWD dataset alone, and (ii) joint training using a combined dataset comprising both SW and R-PWD images. These experiments are designed to assess YOLO-PTHD’s robustness across distinct tree diseases and varying imaging scenarios.

For the main comparative study, five YOLO models from different release stages were selected, including YOLOv8 [49], YOLOv9 [53], YOLOv10 [54], YOLOv11 [48], and YOLOv12 [55]. Among them, YOLOv8 and YOLOv11 were released by Ultralytics, while YOLOv12 represents the most recent iteration in the YOLO series. To ensure a fair comparison, all models were trained using identical hyperparameters and on the same datasets. Notably, YOLOv9 was trained using the “t” (tiny) model variant, while the others used the “n” (nano) variant, indicating that lightweight configurations were employed across all models for consistency and deployment feasibility.

### 3.1. Evaluation of the Performance of YOLO-PTHD Using P-R Curves

**Figure 10:**
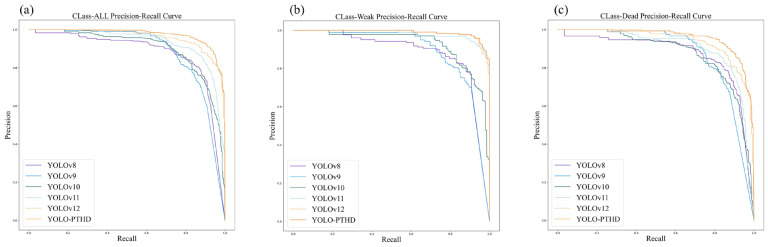
The precision–recall (PR) curves for different models across three categories: (**a**) Class_SWAll category; (**b**) Class_Weak category; (**c**) Class_Dead category.

As illustrated in Figure 10, YOLO-PTHD achieved the highest performance across all three categories, particularly in Class_SWAll and Class_SWWeak. Figure 10a shows that YOLO-PTHD achieved the most balanced accuracy-recall trade-off in identifying target pine trees across both categories. Figure 10b shows that for detecting weakened pine trees, YOLO-PTHD maintains a notable lead over the state-of-the-art YOLOv12. Figure 10 indicates that for detecting dead pine trees, YOLO-PTHD exhibits a greater advantage over other models.

### 3.2. Performance Evaluation

The performance of YOLO-PTHD was evaluated and compared against five YOLO baseline models using the Sirex Woodwasp dataset. Table 3 presents detailed quantitative results across three evaluation metrics—mAP and F1-score for three target categories: Class_SWAll Class_SWWeak, and (Class_SWDead).

YOLO-PTHD achieved the highest performance among all evaluated YOLO models across multiple detection categories. Compared with YOLOv12, it showed a 2.9% improvement in mAP and a 3.2% increase in F1-score for overall detection Class_SWAll. For weakened trees (Class_SWWeak), mAP and F1-score increased by 2.7% and 3.0%, respectively. The most notable gains were observed in the detection of dead trees (Class_SWDead), where mAP improved by 3.1% and F1-score by 3.4%. These results indicate that YOLO-PTHD delivers more reliable identification of both early and advanced symptoms of pine decline.

**Figure 11:**
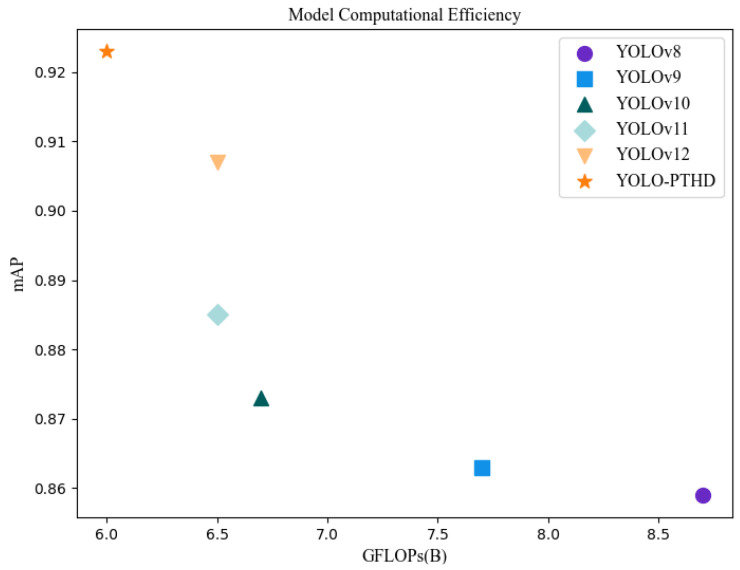
Computational efficiency of various models.

In terms of computational complexity, YOLO-PTHD demonstrated strong efficiency. As shown in Table 3, it required 6.0 GFLOPs, representing a 7.69% reduction compared to YOLOv12 and a 31.03% reduction compared to YOLOv8 (8.7 GFLOPs). Figure 11 visualizes the trade-off between accuracy (mAP) and computational cost (GFLOPs), confirming that YOLO-PTHD achieves superior detection accuracy with the lowest computational demand among all models tested.

### 3.3. Ablation Study

**Figure 12:**
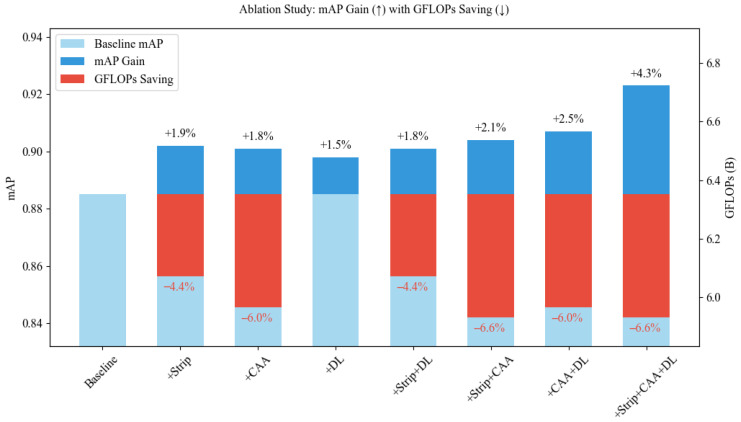
Ablation analysis of accuracy improvement and computational cost reduction.

To assess the contribution of each proposed module, ablation experiments were conducted based on YOLOv11. As shown in Figure 12 the Strip, CAA, and DL modules individually improved mAP by 1.9%, 1.8%, and 1.5%, respectively. Pairwise combinations such as Strip + CAA, CAA + DL, and Strip+DL yielded further gains, while the full model (YOLO-PTHD) achieved the highest mAP improvement of 4.3%.

In terms of computational efficiency, the Strip and CAA modules reduced GFLOPs by 4.4% and 6.0%, respectively, and their combination achieved a maximum reduction of 6.6%. The DL module did not affect GFLOPs but contributed to accuracy.

These results indicate that each module contributes to detection performance, with Strip and CAA also offering efficiency benefits.

These results confirm that each module helps improve detection performance, and Strip and CAA are also effective in reducing computational cost.

To offer a clearer understanding of the three modules’ impact on detection performance, the ablation study outcomes were visualized. Using Grad-CAM [56], the highlighted image regions where the model focuses for predictions are visually illustrated.

**Figure 13:**
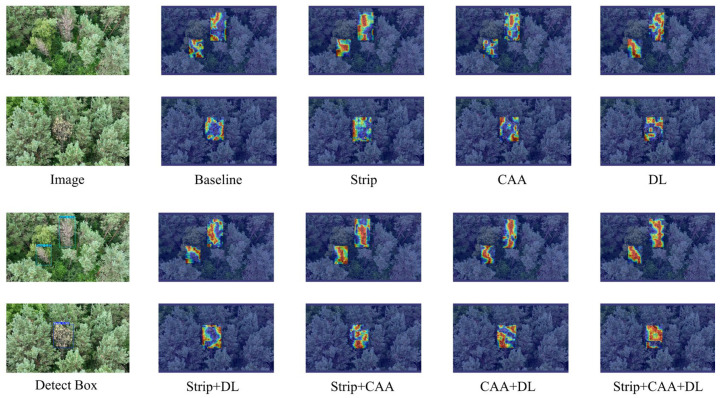
Visualization of ablation study.

The left-side images show the original images alongside those annotated with detection bounding boxes. The upper image presents a scene with two dead pine trees, whereas the lower image shows one weakened pine tree. All models successfully recognized these scenes, as illustrated in Figure 13.

The heatmaps in Figure 13, where brighter colors signify higher model focus, illustrate how the model utilizes features from specific regions for detection. The baseline demonstrates that without these modules, the model fails to accurately focus on needle and canopy characteristics.

In contrast, incorporating either the Strip or CAA module significantly enhances the model’s focus on the needles and canopy. The combined effect of both modules nearly fully directs the model’s focus to the needles and canopy. The DL module enhances the model’s focus on small-area canopy and needle features but requires combination with Strip and CAA for optimal performance.

### 3.4. Validation and Application of YOLO-PTHD in Ground Survey

The georeferenced coordinates of 141 UAV orthophotos covering Plot A were imported into the GIS environment, after which the YOLO-PTHD model was executed to detect weakened and dead pine trees. Only bounding boxes with a confidence score greater than 0.60 were retained to minimize false positives. The detection process was conducted under the configuration described in Section 2.3.4 and required approximately 5 h to complete. In the visualization, weakened trees were rendered as yellow circles and dead trees as white triangles.

**Figure 14:**
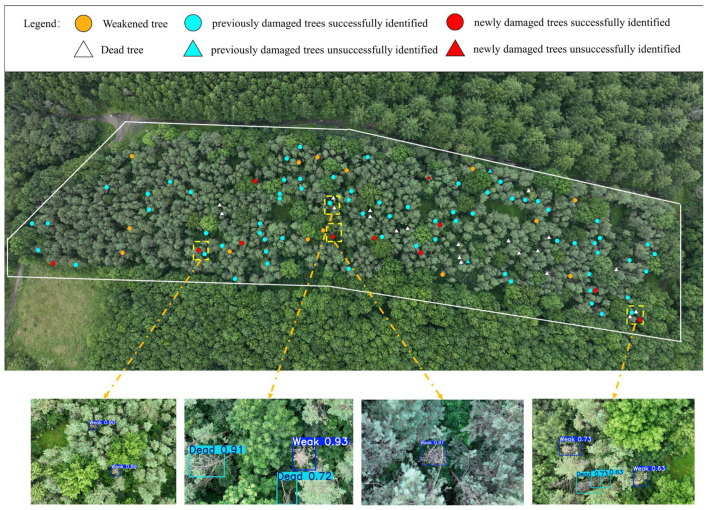
Survey results and model detection outcomes for Plot A. The results of the ground survey and the results of the YOLO-PTHD identification are labeled.

To verify model performance, these detections were compared with a comprehensive ground survey that distinguished two classes of Sirex woodwasp damage: (i) trees damaged in previous years and (ii) trees newly damaged during the current survey. Spatial overlap between survey points and model predictions determined detection success. For previously damaged trees, successful matches were re-labeled with blue circles; unmatched trees were marked with blue triangles. For newly damaged trees, successful detections were shown as red circles, whereas omissions were indicated with red triangles. The resulting composite map (Figure 14) provides an intuitive overview of the health status and highlights areas where the model succeeded or failed. Representative image chips beneath the main panel further illustrate that the predicted bounding boxes generally align well with field observations, even in scenes containing multiple weakened or dead trees.

YOLO-PTHD detected 68 of the 70 previously damaged trees and 10 of the 11 newly damaged trees recorded during the ground survey, yielding accuracy rates of 97.14% and 90.91%, respectively. These results, summarized in Table 4, indicate that the model achieved an overall detection accuracy of 96.30% in real forest conditions. This strong agreement between ground survey and model outputs confirms YOLO-PTHD’s effectiveness in identifying Sirex woodwasp damage with high reliability and low omission risk.

### 3.5. Generalization Capability of the Model

To evaluate the robustness and cross-domain adaptability of YOLO-PTHD, we conducted experiments under two settings: (1) training on the R-PWD dataset to assess generalization to unseen symptom patterns and imaging domains, and (2) joint training on a merged dataset combining different tree diseases (SW + R-PWD) to evaluate cross-disease generalization performance.

#### 3.5.1. Generalization Performance on the R-PWD Dataset

The Real Pine Wilt Disease (R-PWD) dataset (Appendix A.1) contains UAV-acquired imagery collected from various forest sites in South Korea, annotated with two symptom classes: Infected and Dead. Unlike the SW dataset used in prior training, R-PWD represents a different disease type and was captured under distinct environmental conditions.

To assess YOLO-PTHD’s ability to generalize beyond its source domain, we trained the model solely on the R-PWD dataset using the same hyperparameter configuration as in previous experiments. Performance metrics are reported in Appendix A Table A2, and compared with the results of EfficientNetv2-S from [47]. YOLO-PTHD achieves higher precision (0.908), recall (0.926), and F1 score (0.917) values, surpassing EfficientNetv2-S in all metrics. This demonstrates that the model can effectively recognize symptoms of pine wilt disease, despite differences in acquisition platform, disease characteristics, and label distributions, thus validating its domain-level generalization capability.

#### 3.5.2. Cross-Disease Detection on the Combined SW + R-PWD Dataset

To further examine the model’s ability to generalize across multiple tree diseases, we constructed a balanced dataset by combining the full SW dataset (1330 images) with a randomly sampled subset of R-PWD (665 images per class). The merged dataset contains 2660 images evenly distributed across four classes: SWWeak, SWDead, PWD-Infected, and PWD-Dead. A stratified 70/30 train/validation split was applied to ensure balanced representation of all classes.

Detection results on the combined SW + R-PWD dataset are presented in Table 5. YOLO-PTHD achieved the best overall performance, with a mAP of 0.918 and an F1-score of 0.888, outperforming all baseline YOLO models. Compared to YOLOv12, YOLO-PTHD showed a 3.7% increase in mAP and a 1.6% gain in F1-score for the overall category (Class_SWPWDAll). Notably, across all four subclasses—including weakened and dead trees in both SW and PWD domains—YOLO-PTHD consistently produced the highest or near-highest scores, reflecting its strong capability in handling different symptom stages and disease types.

These results demonstrate that the proposed model effectively captures transferable visual patterns associated with pine tree decline, supporting its robust cross-disease generalization on heterogeneous UAV imagery from different geographic and pathological contexts.

## 4. Discussion

This study proposed YOLO-PTHD, a lightweight yet high-performance deep learning framework designed for UAV-based detection of pine tree health conditions under various biotic stressors. Validated on two distinct datasets—covering both Sirex woodwasp-induced damage and pine wilt disease—the model demonstrated strong generalization across different pest types, geographic locations, and imaging conditions. YOLO-PTHD achieved an overall detection accuracy of 96.3% in field-verified *Sirex* outbreak areas and outperformed five state-of-the-art YOLO variants with a mAP of 0.923, an F1-score of 0.866, and a reduced computational cost of 6.0 GFLOPs. Through the integration of Strip-based convolution, Channel-Aware Attention, and a scale-sensitive dynamic loss function, YOLO-PTHD effectively addresses critical challenges in phenotype-level detection of pine decline, such as subtle needle discoloration, elongated canopy structures, and occlusion in dense forest environments. These results confirm the model’s robustness, efficiency, and practical value as a scalable tool for forest health surveillance, early pest outbreak detection, and ecological risk mitigation.

Our findings are consistent with previous studies emphasizing the utility of deep learning in tree health monitoring. In prior work, DeepForest and YOLOv5 were jointly applied to detect bark beetle damage in Mexican pine forests, with detection primarily based on visibly discolored canopies [16]. Another study developed the YOLO-PWD model for pine wilt disease identification, incorporating attention mechanisms to improve accuracy, yet focusing mainly on clearly dead or severely affected trees [17]. In contrast, YOLO-PTHD demonstrates improved sensitivity to visually detectable symptoms such as needle yellowing and crown thinning. This may be attributed to the StripBlock-enhanced backbone and CAA-integrated neck, which enable extraction of orientation-aware and context-rich features suited to pine crown morphology. As shown in the ablation study, each proposed module—Strip, CAA, and SDIoU—independently contributed to performance gains, with a combined mAP improvement of 4.3%. Grad-CAM visualizations (Figure 13) further illustrate enhanced model attention on needle and canopy features, supporting more accurate detection under complex forest conditions.

In comparison with previous studies on Sirex woodwasp detection, which primarily relied on multispectral imagery and traditional classifiers such as Random Forest and SVM [26,27], YOLO-PTHD offers clear advantages in model adaptability and operational efficiency. First, it reduces reliance on handcrafted spectral features by leveraging end-to-end deep learning from RGB UAV imagery, enabling early identification of subtle phenotypes such as crown thinning and needle yellowing. Second, the object detection framework allows for more efficient annotation at the crown level, greatly accelerating dataset development. Experiments on the SW dataset confirm YOLO-PTHD’s strong detection capability for Sirex woodwasp damage. When retrained on the R-PWD dataset, the model maintained high accuracy under different forest types and imaging conditions, demonstrating its cross-region and cross-pest generalization. Furthermore, training on the combined SW and R-PWD dataset showed that the model could distinguish between different crown phenotypes associated with Sirex woodwasp and pine wilt disease, highlighting its potential as a unified deep learning backbone for monitoring multiple forest health threats in *Pinus* ecosystems.

Despite its strong performance, YOLO-PTHD has key limitations that warrant consideration. Most notably, the model relies solely on RGB imagery, which restricts its ability to detect early physiological stress before visible symptoms appear. Moreover, multiple biotic and abiotic factors such as drought, fungal pathogens, and nutrient deficiencies can cause phenotypic changes like needle yellowing and crown thinning that resemble symptoms induced by Sirex woodwasp. Consequently, while YOLO-PTHD can effectively detect declining trees and is well suited for monitoring in areas with confirmed Sirex woodwasp outbreaks, it cannot independently confirm pest-specific damage in regions where the cause of decline is unknown. In such cases, RGB-based detection must be supplemented with additional evidence [57] to accurately attribute decline to Sirex woodwasp.

On the deployment side, YOLO-PTHD (6.0 GFLOPs) achieves a lower computational load compared to YOLOv11n (6.5 GFLOPs), making it well suited for real-time inference on lightweight edge devices such as the Jetson Nano [58]. This efficiency is particularly valuable in field operations. For example, in this study, UAV flights over Plot A required approximately 90 min, and the YOLO-PTHD model processed all acquired images within 5 h. In contrast, the corresponding ground survey conducted by six trained researchers took 12 full days to complete the same area. These results highlight the model’s potential to substantially reduce the time and labor required for forest health assessments. By enabling rapid onboard analysis during UAV flights, deployment on edge devices can further minimize data transfer delays and accelerate detection of tree decline. To enhance this capability, future work could explore model pruning or knowledge distillation techniques to further reduce inference time and resource demand, supporting real-time forest monitoring and precision pest management in operational settings.

## 5. Conclusions

This study presents YOLO-PTHD, a lightweight deep learning model tailored for UAV-based detection of pine-tree health under biotic stresses. Trained and evaluated on the newly constructed Sirex Woodwasp (SW) dataset, the model achieved 96.3% overall accuracy, mAP 0.923, and F1-score 0.866 while requiring only 6.0 GFLOPs—outperforming five state-of-the-art YOLO baselines in both accuracy and efficiency. Ablation experiments confirmed that each targeted innovation—StripBlock convolution, Channel-Aware Attention, and the scale-adaptive SDIoU loss—contributes incrementally to performance, yielding a combined 4.3% mAP gain and a 6.6% reduction in computation relative to the YOLOv11 backbone.

Robustness tests demonstrated strong generalization. When retrained on the independent Real Pine Wilt Disease (R-PWD) dataset, YOLO-PTHD reached precision 0.908, recall 0.926, and F1-score 0.917, surpassing a recently reported EfficientNetv2-S benchmark [47]. On the combined SW + R-PWD dataset, the model attained mAP 0.918 and F1-score 0.888, accurately distinguishing crown phenotypes produced by two distinct pests and validating its cross-disease adaptability.

By combining sensitivity to phenotypic indicators of tree decline with computational efficiency, YOLO-PTHD serves as a practical and scalable tool for large-scale forest health surveillance and rapid pest outbreak response. Its tree-level annotation workflow accelerates dataset expansion, and its compact footprint makes deployment on edge devices (e.g., Jetson-class modules or UAV onboard processors) feasible. These strengths make YOLO-PTHD a scalable foundation for multi-disease surveillance in *Pinus* ecosystems and a promising component of real-time, precision pest management systems. By enabling accurate and timely detection of pest-induced decline, the model can support responsive forest management and facilitate early intervention in pest mitigation efforts.

## Figures and Tables

**Table 1:** Image statistics before and after preprocessing.

Plot	Original Image	Preprocessed Image
A	443	353
B	398	263
C	572	449
D	338	265

**Table 2:** Model training hyperparameter settings.

Parameter Name	Value
Input size	640 × 640
Learning rate	0.01
Batch size	16
Epoch	300
Momentum	0.8
Optimizer	SGD

**Table 3:** Comparative performance of models.

Model	mAP			F1			GFLOPs
	SWAll	SWWeak	SWDead	SWAll	SWWeak	SWDead	
YOLOv8	0.859	0.871	0.846	0.801	0.814	0.787	8.7
YOLOv9	0.863	0.873	0.853	0.813	0.826	0.799	7.7
YOLOv10	0.873	0.885	0.860	0.825	0.838	0.811	6.7
YOLOv11	0.885	0.898	0.872	0.837	0.851	0.823	6.5
YOLOv12	0.897	0.91	0.884	0.839	0.853	0.825	6.3
YOLO-PTHD	0.923	0.935	0.911	0.866	0.879	0.853	6.0

**Table 4:** Number of weakened, dead, and damaged trees identified by survey and model.

Category	Sum	Weak/Detected ^1^	Missed by Model ^2^
Model detected Weak	92	78	-
Model detected Dead	20	-	-
Previously damaged trees	70	68	2
Newly damaged trees	11	10	1

^1.^ The ground survey found the same number of damaged trees as the model detected weakened trees. ^2.^ During the ground survey, damaged trees were identified that the model failed to successfully detect.

**Table 5:** Performance on the SW + R-PWD dataset.

Model	mAP					F1				
	SWPWDAll	SWWeak	SWDead	PWDInfected	PWD Dead	SWPWDAll	SWWeak	SWDead	PWDInfected	PWD Dead
YOLOv8	0.849	0.849	0.854	0.848	0.846	0.84	0.839	0.854	0.828	0.838
YOLOv9	0.861	0.861	0.861	0.865	0.852	0.849	0.852	0.863	0.821	0.861
YOLOv10	0.863	0.863	0.865	0.866	0.858	0.852	0.853	0.851	0.842	0.863
YOLOv11	0.877	0.876	0.88	0.882	0.869	0.858	0.861	0.844	0.874	0.852
YOLOv12	0.885	0.891	0.876	0.892	0.881	0.874	0.883	0.862	0.886	0.865
YOLO-PTHD	0.918	0.921	0.905	0.932	0.914	0.888	0.9	0.881	0.874	0.897

## Data Availability

The dataset for pine wilt disease can be obtained from the following URL: https://doi.org/10.1016/j.compag.2024.108690 (accessed on 28 February 2025).

## Abbreviations

The following abbreviations are used in this manuscript:

**Abbreviation**	**Full Term**	**Explanation**
YOLO	You Only Look Once	A deep learning algorithm for fast object detection.
C3k2	Cross-stage Convolutional Block with two branches	A fast convolutional block with optional C3k or Bottleneck modules.
Strip	StripBlock Module	A directional convolution module used to extract long-range features along vertical and horizontal axes.
CAA	Channel-Aware Attention Module	An attention module that applies directional weights to improve detection of elongated or occluded features.
DL	Dynamic Loss	A scale-sensitive loss function that adjusts based on target size to improve detection of small or irregular objects.
Strip-C3k2	Strip-enhanced C3k2 block	A variant of C3k2 that uses directional strip convolutions to better detect elongated tree structures.
CAA-C3k2	Channel-Aware Attention enhanced C3k2 block	A C3k2 block with attention mechanisms to enhance weak visual signals such as early tree decline.
C2PSA	Convolutional 2-layer with PSA Attention	A two-layer block that integrates Pyramid Squeeze Attention for better features.
DWconv	Depthwise Convolution	A lightweight convolution operation that reduces computational cost while preserving features.
Conv	Convolution	A fundamental operation in neural networks used to extract features from images.
SPPF	Spatial Pyramid Pooling Fast	A fast pooling module that extracts multi-scale features with reduced overhead.
Upsample	Upsample	An operation that increases feature map resolution to align layers for feature fusion.
Concat	Concatenation	The process of merging feature maps from different layers to enhance detection performance.
Detect	Detection Head	The final component of the network that outputs object categories and bounding box locations.
x_1_ (Section 2.3.1)	StripBlock Scaling Coefficient	A learnable scaling parameter in StripBlock to stabilize output during training.
x_2_ (Section 2.3.2)	Direction-aware Channel Weight	A channel-wise weight in CAA used to enhance direction-specific feature representation.
IoU	Intersection over Union	A metric used to evaluate the overlap between predicted and ground truth bounding boxes in object detection.
Hyperparameter	Hyperparameter	A predefined configuration value (e.g., learning rate, batch size) that governs model training but is not learned by the model.
SW	Sirex Woodwasp	*Sirex noctilio (Hymenoptera: Siricidae)*
PWD	Pine Wilt Disease	Refers to tree decline caused by pine wilt disease, typically induced by *Bursaphelenchus xylophilus.*
SWPWDAll	All Trees in Sirex Woodwasp and Pine Wilt Disease Datasets	All trees classified in the Sirex Woodwasp (SW) and Pine Wilt Disease (PWD) datasets: SWWeak, SWDead, PWDInfected, and PWDDead.
SWAll	All Trees in Sirex Woodwasp Dataset	All trees classified in the SW dataset: SWWeak and SWDead.
SWWeak	Weakened Trees in Sirex Woodwasp Dataset	Trees classified in the SW dataset that show early symptoms of decline, such as yellowing needles and sparse canopies.
SWDead	Dead Trees in Sirex Woodwasp Dataset	Trees classified in the SW dataset that are dead due to Sirex woodwasp, with no green needles.
PWDInfected	Infected Trees in Pine Wilt Disease Dataset	Trees classified in the PWD dataset showing early infection symptoms such as yellowing or browning.
PWDDead	Dead Trees in Pine Wilt Disease Dataset	Trees classified in the PWD dataset that are dead due to pine wilt disease, with defoliation or severe discoloration.

## Appendix A

This appendix presents an evaluation of the proposed YOLO-PTHD model on the Real Pine Wilt Disease (R-PWD) dataset. The purpose of this experiment is to assess the generalization capability of YOLO-PTHD when applied to plant disease datasets beyond its training domain. PWD was selected as a representative test case to examine the model’s ability to recognize symptoms of a different plant disease under real-world UAV imaging conditions.

To contextualize the performance of YOLO-PTHD, we include a reference comparison with results reported in [47], which utilized the EfficientNetv2-S model for classifying PWD symptoms. It is important to note that EfficientNetv2-S was not re-implemented in this study; its evaluation metrics are cited directly from the published work and are used for comparison purposes only.

### Appendix A.1. Real Pine Wilt Disease Dataset

The R-PWD dataset was collected in various forest regions across South Korea using UAV platforms during the PWD observation season (August to October). Videos were captured at flight altitudes ranging from 50 to 70 m using DJI Mavic 2 Pro and custom-built UAVs. Still images were extracted and cropped around tree crowns to a fixed resolution of 300 × 300 pixels. The overall composition of the dataset is summarized in Table A1.

All images were manually annotated using the open-source tool LabelImg [46], following a consistent labeling protocol. Only two classes were considered:

1. Infected: Trees showing initial signs of infection, such as yellowing or browning of foliage.
2. Dead: Trees that have lost foliage or exhibit severe desiccation and grayish discoloration.

The annotated dataset was randomly split into training (70%) and validation (30%) subsets, ensuring a balanced distribution of both classes in each split.

**Table A1:** Composition of the Real PWD (R-PWD) dataset.

Location	Infected	Dead
Sangsaek-ri	282	276
Hasaek-ri	359	212
Gaegok-ri	306	343
Jicheon-myeon	327	320
Hadong/Geochang-gun	97	201
Total	1371	1352

### Appendix A.2. Model Training and Performance Comparison

The YOLO-PTHD model was trained on the annotated R-PWD dataset using the data split described in Appendix A.1. Training followed the hyperparameter configuration listed in Table 2 of the main paper. Evaluation was conducted on the validation set. For comparison, we include the reported results of EfficientNetv2-S from [47], which was used for the same task.

As shown in Table A2, YOLO-PTHD achieves higher precision, recall, and F1 score values compared to the reported results of EfficientNetv2-S in [47]. This demonstrates the model’s strong generalization ability when applied to a different plant disease dataset, highlighting its robustness in detecting unseen disease patterns under UAV imagery.

**Table A2:** Performance metrics on the R-PWD dataset.

Model	Dataset	Precision	Recall	F1
EfficientNetv2-S [47]	R	0.8995	0.9071	0.9022
YOLO-PTHD (ours)	R	0.908	0.926	0.917
